# 

**Published:** 1922-06

**Authors:** 


### CONTENTS.

| | Page |
|---|---|
| **Medicine and Psychology.** By SIR JOHN HERBERT PARSONS, C.B.E., F.R.C.S., F.R.S. | 1 |
| **Discussion on Renal Efficiency.** By CECIL CLARKE, M.D. Lond., M.R.C.P., J. O. SYMES, M.D. Lond., G. HADFIELD, M.D. Lond. | 13 |
| **A Physiological Conception of Prolonged Hysterical Conditions.** By H. H. CARLETON, M.A., M.D. Oxon. | 36 |
| **Reviews of Books** | 47 |
| **Editorial Notes** | 52 |
| **Obituary:** ROBERT SHINGLETON SMITH, M.D., B.Sc., F.R.C.P. | 57 |
| **Local Medical Notes** | 67 |

